# Prolyl Hydroxylase PHD3 Enhances the Hypoxic Survival and G1 to S Transition of Carcinoma Cells

**DOI:** 10.1371/journal.pone.0027112

**Published:** 2011-11-08

**Authors:** Heidi Högel, Krista Rantanen, Terhi Jokilehto, Reidar Grenman, Panu M. Jaakkola

**Affiliations:** 1 Turku Centre for Biotechnology, Turku University and Åbo Akademi University, Turku, Finland; 2 Department of Medical Biochemistry, Turku University, Turku, Finland; 3 Department of Otorhinolaryngology, Head and Neck Surgery, Turku University Hospital, Turku, Finland; 4 Department of Oncology and Radiotherapy, Turku University Hospital, Turku, Finland; Istituto Dermopatico dell'Immacolata-IRCCS, Italy

## Abstract

Hypoxia restricts cell proliferation and cell cycle progression at the G1/S interface but at least a subpopulation of carcinoma cells can escape the restriction. In carcinoma hypoxia may in fact select for cells with enhanced hypoxic survival and increased aggressiveness. The cellular oxygen sensors HIF proline hydroxylases (PHDs) adapt the cellular functions to lowered environmental oxygen tension. PHD3 isoform has shown the strongest hypoxic upregulation among the family members. We detected a strong PHD3 mRNA expression in tumors of head and neck squamous cell carcinoma (HNSCC). The PHD3 expression associated with expression of hypoxic marker gene. Using siRNA in cell lines derived from HNSCC we show that specific inhibition of PHD3 expression in carcinoma cells caused reduced cell survival in hypoxia. The loss of PHD3, but not that of PHD2, led to marked cell number reduction. Although caspase-3 was activated at early hypoxia no induction of apoptosis was detected. However, hypoxic PHD3 inhibition caused a block in cell cycle progression. Cell population in G1 phase was increased and the population in S phase reduced demonstrating a block in G1 to S transition under PHD3 inhibition. In line with this, the level of hyperphosphorylated retinoblastoma protein Rb was reduced by PHD3 knock-down in hypoxia. PHD3 loss led to increase in cyclin-dependent kinase inhibitor p27 expression but not that of p21 or p16. The data demonstrated that increased PHD3 expression under hypoxia enhances cell cycle progression and survival of carcinoma cells.

## Introduction

Oxygen level in tissues is reduced in many pathological conditions including cancer progression [1]. Solid tumours develop areas of poor oxygenation as they outgrow their blood supply. Mammalian cells have developed an array of mechanisms in order to adapt to and survive under varying oxygen tensions. The responses to hypoxia include upregulation of angiogenic factors and the switch from oxidative to glycolytic metabolism [2]. In cancer cells low oxygen tension induces genetic instability [3], [4] and is a strong driving force in the clonal selection that supports more aggressive disease.

When cells are exposed to acute hypoxia cell proliferation is inhibited by several mechanisms such as activation of quiescence, apoptosis, necrosis and differentiation. In hypoxia also cell cycle is arrested occuring at the G1/S interface [5], [6]. The key molecular event needed for the G1 to S transition is the hyperphosphorylation of the retinoblastoma protein (Rb) that is performed by cyclin-dependent kinase (CDK) –cyclin complexes. Hyperphosphorylation of Rb at late G1 releases E2F transcription factor and allows S-phase to proceed. Under hypoxic conditions Rb is hypophosphorylated thus inhibiting the transcription of S-phase genes and cell cycle progression [7], [8], [9]. Several possible mechanisms as to how hypoxia may regulate cell cycle progression have been suggested. Hypoxia has been reported to upregulate p21(Cip1/Waf1/CDKN1A) and p27(Kip1/CDKN1B) which regulate the inactivation of cyclin E – CDK2 –complex. While there is some controversy on the role of p27, the hypoxic cell cycle arrest does not seem to require p21 [10], [11]. Hypoxia may also activate cell cycle arrest by inhibiting c-Myc transcriptional activity [12]. Also p16(INK4a) that attenuates cell cycle progression has been shown to be hypoxia-inducible [13]. Noticeably, a subpopulation of cancer cells need to escape the hypoxic cell cycle arrest in order to maintain growth.

Many of the responses to hypoxia are mediated by hypoxia-inducible transcription factor (HIF) that is rapidly degraded in normoxia but stabilized under hypoxia [14]. In normoxia the alpha subunit (HIF-α) is hydroxylated by a family of prolyl hydroxylase domain containing enzymes (PHDs) [15], [16]. Hydroxylation of HIF-α allows its recognition by the von Hippel-Lindau protein and leads to subsequent proteosomal degradation of HIF-α [17], [18], [19], [20]. PHDs are enzymes that require molecular oxygen as substrate. Therefore under hypoxic conditions the activity of PHDs is lowered thus allowing HIF-α to escape degradation. The stabilized HIF-α is translocated to nucleus where it dimerizes with HIF-1beta forming an active transcription factor complex that activates several target genes that may include those regulating cell cycle [21].

In mammals three prolyl hydroxylase isoforms termed PHD1, PHD2 and PHD3 (also called EGLN2, EGLN, EGLN3, respectively) have been characterized [22]. All of these isoforms have been shown to hydroxylate HIF-α *in vitro* and to have similar co-factor requirements. The most ubiquitous factor controlling the activity of these enzymes is oxygen concentration but they also require 2-oxoglutarate and iron as cofactors. Despite the similarities in their requirements, several differences in their function and characteristics exist [23]. Unlike the other two isoforms, PHD3 has been shown to cause apoptosis in neuronal cells as well as in cancer cells when expressed under normoxia at high levels [24], [25], [26], [27]. PHD3 is also the isoform that shows most robust induction under hypoxia [28], [29], [30], [31]. The elevated expression is likely to at least partially compensate for the reduced activity under hypoxia. In fact, PHD3 is known to retain much of its enzymatic activity at least under moderate hypoxia [32].

Here we have studied the role of PHD3 depletion under hypoxia in cancer cell lines. We show that PHD3 is upregulated in squamous cell carcinoma tumours. We show that PHD3 depletion under hypoxia leads to cell cycle arrest that occurs at the G1/S boundary. In line with this, the depletion of PHD3 reduces the amount of hyperphosphorylated retinoblastoma protein as well as the amount of cyclin D1. Moreover, PHD3 inhibition upregulates the expression of cyclin-dependent kinase inhibitor p27.

## Results

### Head and neck squamous cell carcinoma tumors overexpress PHD3

**Figure 1:**
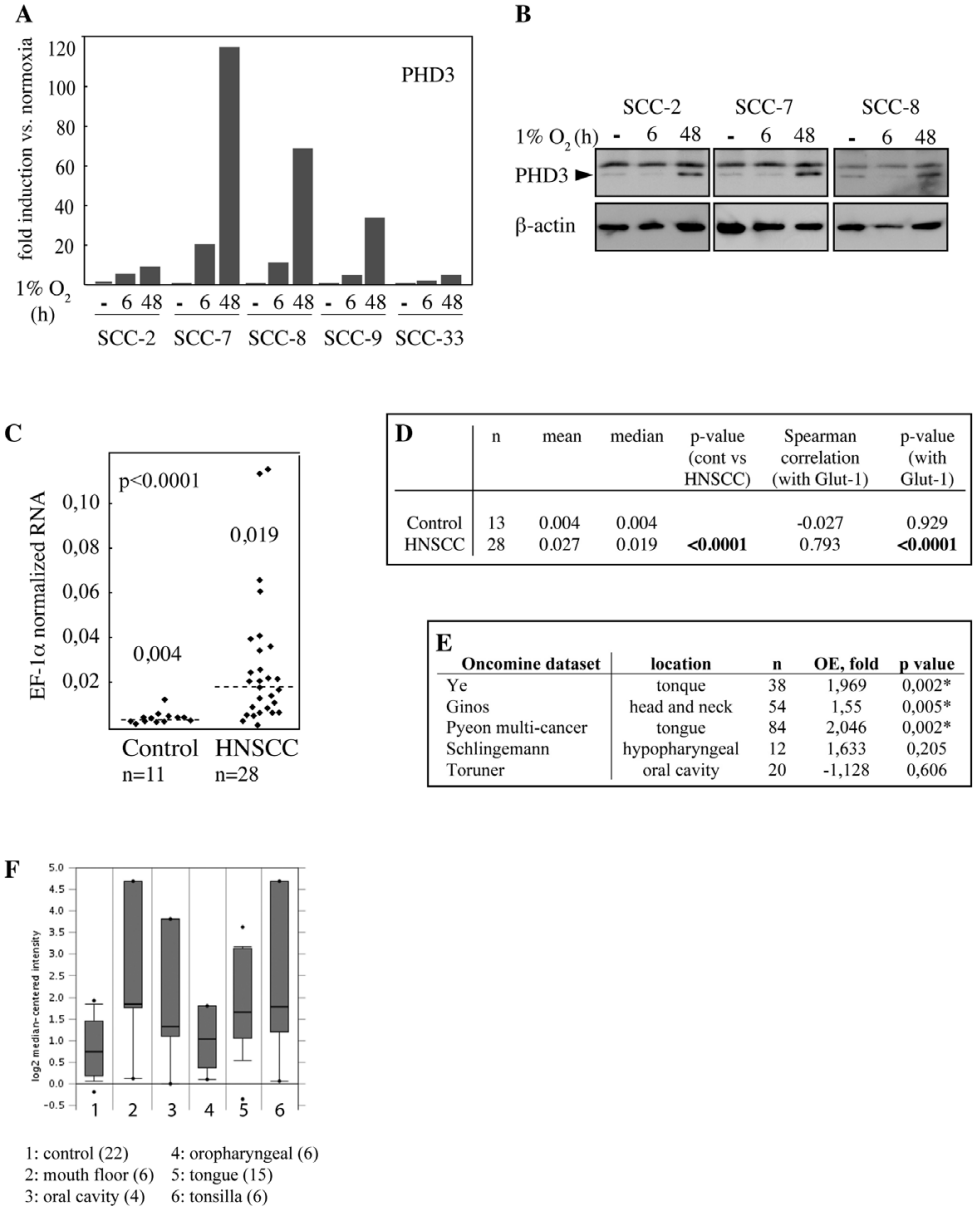
PHD3 is overexpressed in human HNSCC and induced by hypoxia. (*A*) Five different primary human head and neck squamous cell carcinoma-derived cell lines (SCC) were cultured in normoxia or in hypoxia for 6 and 48 hours. The PHD3 mRNA expression was detected and quantified by Q-RT-PCR. (*B*) PHD3 expression in three different SCC cell lines detected by western blot analysis. (*C*) PHD3 mRNA expression in 28 HNSCC patient tumour samples and in 11 noncancerous anatomically matching samples was determined by Q-RT-PCR and normalized to EF-1α levels in each sample. Each dot represents the expression in one sample. The median values are indicated by dashed line and numerical value. (*D*) The statistical significance of PHD3 mRNA expression in patient and control samples was determined using Spearman correlation and two-tailed Student's t-test. The upregulation of PHD3 gene shows statistical significance (p-value<0,0001) and correlates with Glut-1 expression (p-value<0,0001). (*E*) Four of five independent head and neck cancer studies extracted from Oncomine database show overexpression of PHD3 in cancer tissue relative to normal tissue. Three of the studies show statistically significant overexpression of PHD3 in cancer samples compared to normal tissue. (*F*) Detailed data analysis extracted from one Oncomine database study [37] for PHD3 expression. The expression is shown in five anatomically different HNSCC sites relative to control samples.

PHD3 has been reported to be activated by hypoxia in several cell types including diverse carcinoma cell lines [30], [33], [34]. In order to study hypoxic PHD3 expression in human head and neck squamous cell carcinoma (HNSCC) we used five different primary cell lines (UT-SCC) established from HNSCC patients at Turku university hospital [35]. The cells were exposed to normoxia or hypoxia for 6 and 48 hours. PHD3 was strongly induced at mRNA level in all cell lines tested with maximal induction seen after 48-hour hypoxia (Fig. 1A, Fig. S1A,B). In line with this, PHD3 was induced at protein level in three cell line tested (Fig. 1B).

To study the PHD3 mRNA expression in human HNSCC, we analyzed tumor samples from 28 HNSCC patients and from anatomically matching samples in 11 healthy controls. The Q-RT-PCR analysis showed a 5-fold overexpression of PHD3 (p-value<0.0001) in the tumor samples as compared to control (Fig. 1C and D). To investigate whether the PHD3 expression associates with hypoxia, we analyzed the correlation between PHD3 and a widely used hypoxic marker glucose transporter Glut-1. Strong correlation between PHD3 and Glut-1 mRNA expression was detected (Spearman correlation 0.8, p-value<0.0001) implying that PHD3 transcription is activated by hypoxia in HNSCC (Fig. 1D).

To further study the clinical occurrence of PHD3 overexpression in HNSCC we analyzed studies deposited in the Oncomine database [36]. From the five studies comparing PHD3 expression in HNSCC and normal tissue four showed overexpression of PHD3. In three studies the HNSCC overexpression was statistically significant (p-value<0,002 to 0,005) (Fig. 1E). Moreover, one study demonstrated increased PHD3 mRNA level in different anatomical locations including tongue, mouth floor and tonsilla (Fig. 1F) [37]. PHD3 overexpression was not restricted to HNSCC (Table S1).

### PHD3 inhibition reduces hypoxic SCC growth

**Figure 2:**
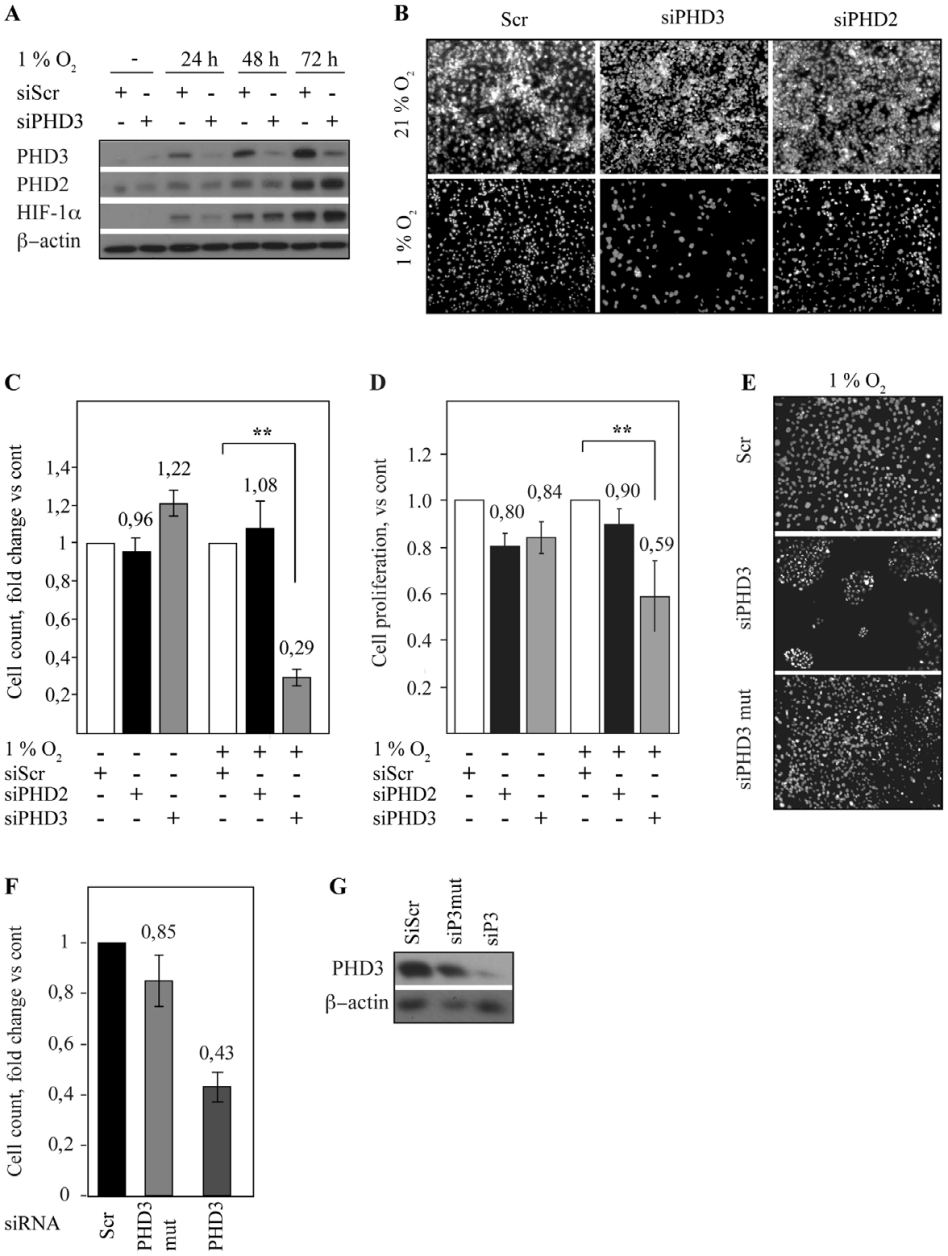
PHD3 is required for SCC cell survival in prolonged hypoxia. (*A*) PHD3 is induced in hypoxia. Knock-down of PHD3 expression with siRNA (siPHD3) is specific and does not affect PHD2 or HIF-1α expression. siScr = scrambled control siRNA. (*B*) SCC2 cells were plated on glass slides, transfected with siRNAs and exposed to hypoxia for 48 h followed by Hoescht nuclear staining. The samples with PHD3 siRNA showed marked reduction in cell number in hypoxia. No effects were seen in the normoxic counterparts. (*C*) Quantification of the cell number in three optical fields from each sample in three independent experiments. The change in cell number is shown as folds vs. control (siScr). The reduction in cell number of siPHD3-transfected samples was statistically significant (**; p-value<0,01). (*D*) The proliferation rate of cells transfected with control (siScr), PHD2 or PHD3 siRNA was determined using BrdU incorporation. Results from absorbance changes in three independent experiments are shown as folds vs. control (siScr). (*E*) To validate the specificity of the effect of PHD3 inhibition on cell survival a point-mutated PHD3 siRNA (siPHD3mut) we used as a control. siPHD3mut did not affect the viability of SCC cells. (*F*) Quantification of the cell number in three optical fields. (*G*) Western blot analysis shows the effect of the indicated siRNAs on PHD3 expression.

As PHD3 was overexpressed in HNSCC we asked whether PHD3 provided growth advantage for the carcinoma cells. We used previously validated double stranded PHD3 siRNA (siPHD3) to knock down the expression of PHD3. As controls non-target (siScr) and PHD2-targeted siRNA (siPHD2) were used [38]. The siRNAs were transfected to UT-SCC2 cells followed by exposure to normoxia (21% O_2_) or hypoxia (1% O_2_) for 48 hours. The PHD3 siRNA was effective and specific as demonstrated by reduced hypoxic PHD3 expression in SCC2 cells (Fig. 2A, Fig. S1C).

Noticeably, we detected that the PHD3 knock-down caused a marked reduction in cell number as compared to controls when grown under hypoxia (Fig. 2B). Quantification of the cell number in hypoxia demonstrated almost 70% reduction in surviving cells after 48-hour hypoxia with siPHD3 as compared to either control or siPHD2 transfected cells (Fig. 2B and C). In line with the fact that little PHD3 expression was seen under normoxia, siPHD3 had no marked effect in normoxia.

In order to verify the specific effect of PHD3 siRNA we used a point-mutated double stranded siRNA (siPHD3mut) as a control. The mutant siRNA did not affect the PHD3 expression. Accordingly, siPHD3mut did not have marked effect on the cell number in hypoxia (Fig. 2E and F) further validating the specific effect of PHD3 knock-down in reducing the number of surviving cells under hypoxia.

We further asked whether the reduction in cell number would be reflected as reduced proliferation rate in siPHD3 cells. PHD3 or control siRNAs were transfected to SCC2 cells followed by BrdU addition and exposure to normoxia or hypoxia for 48 hours. Indeed, BrdU incorporation was reduced in siPHD3 cells as compared to control indicating reduced cell growth under hypoxia and PHD3 inhibition (Fig. 2D).

**Figure 3:**
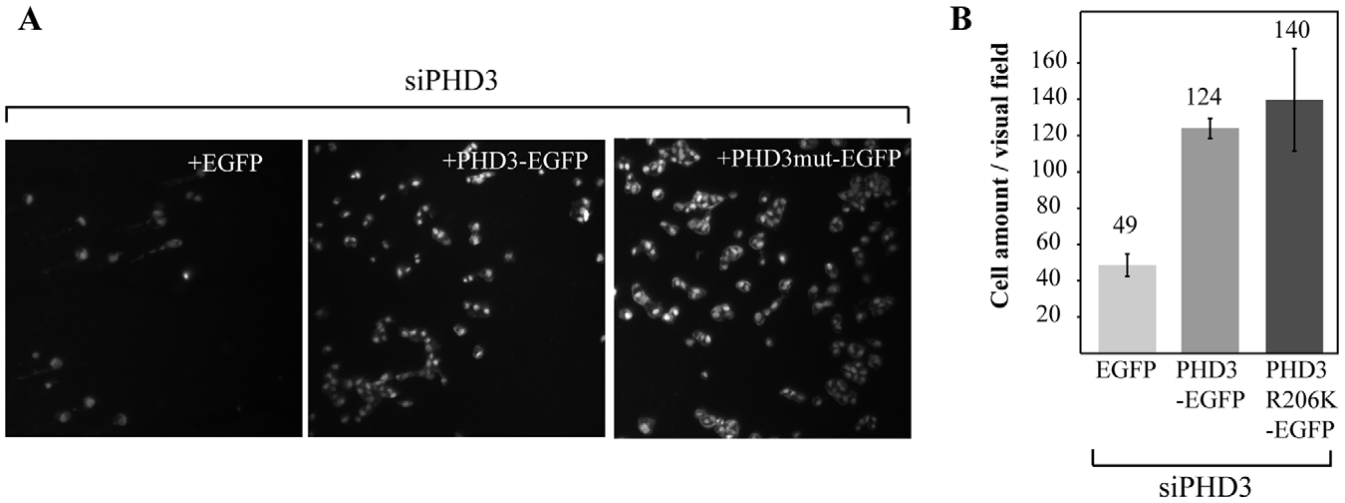
PHD3 hydroxylase activity is not required for hypoxic cell survival. (*A*) Cells were exposed to siPHD3, followed by transfection of the indicated control (EGFP) and PHD3 expressing plasmids (PHD3-EGFP and PHD3R206K-EGFP) and 48 hour incubation in hypoxia. The phase contrast images show representative examples of cell amount. (*A*) Quantification of cell number with transfection of indicated plasmids under siPHD3 expression and hypoxia. Means and SD of three visual fields.

Finally, using a rescue experiment we studied whether the hydroxylase activity of PHD3 was required for supporting hypoxic cell survival. HeLa cells exposed to siPHD3 were transfected with PHD3-EGFP, PHD3(R206K)-EGFP (a hydroxylase-inactive mutant) [26] or EGFP as control followed by two day hypoxic exposure (Fig. 3). The transfection efficiency was controlled with confocal microscopy for EGFP expression (Fig. S2). Cells transfected with PHD3-EGFP demonstrated clearly increased survival as compared to control. Interestingly, PHD3(R206K)-EGFP displayed comparable enhancement in cell number suggesting that hydroxylase activity is not required for PHD3 supported cell survival.

### Inhibition of PHD3 does not activate significant apoptosis in SCC cells

**Figure 4:**
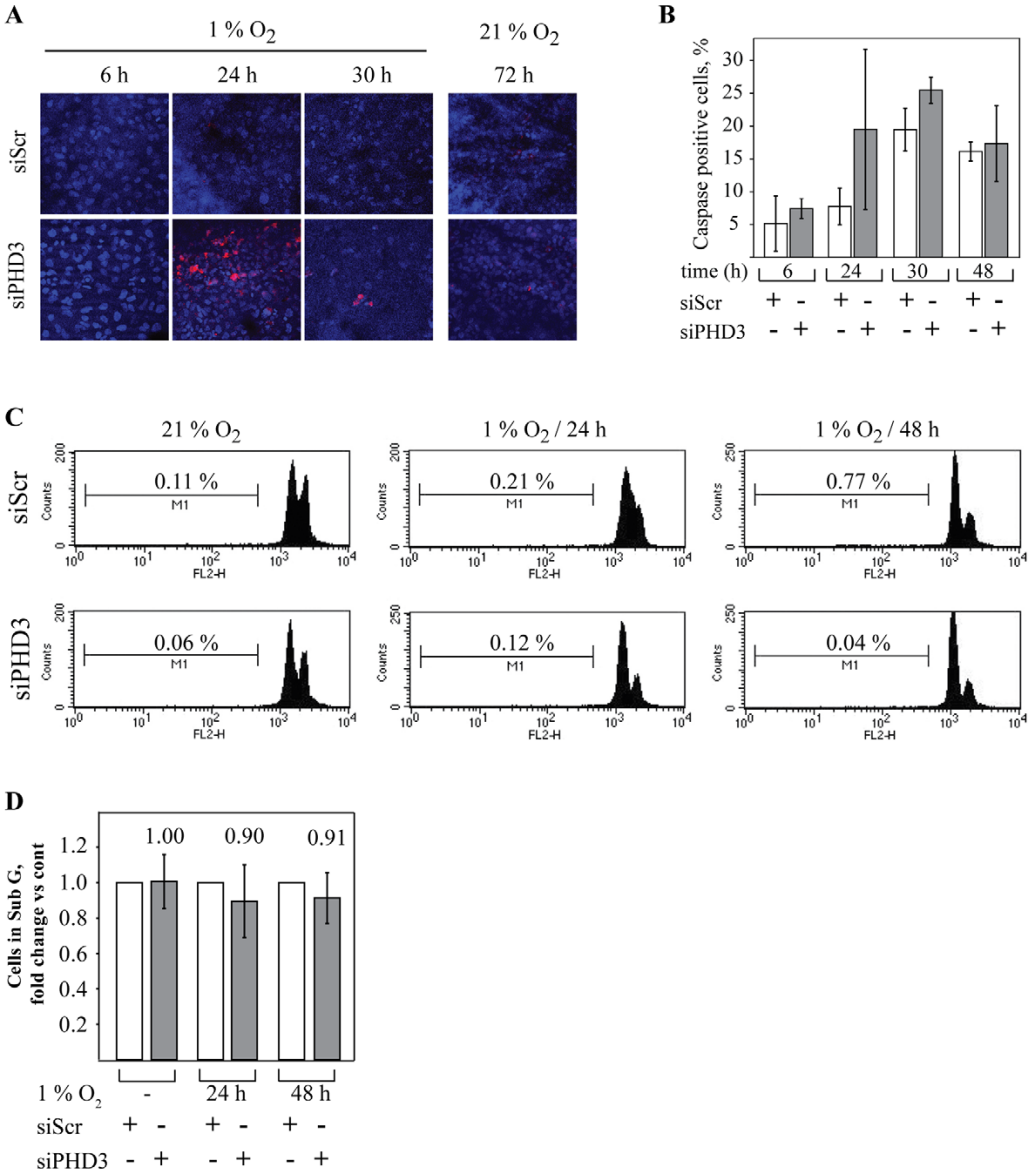
siPHD3 does not activate marked apoptosis. (*A*) SCC2 cells transfected with the indicated siRNAs were exposed to hypoxia for 6 to 30 hours. Cells were subsequently stained for caspase-3 (red) and Hoechst nuclear stain (blue). In siPHD3-transfected cells increased activation of caspase-3 was detected after 24 hour hypoxia. (*B*) Quantification of caspase-3 activation at the indicated hypoxic time points. Means and SD for five visual fields are shown. (*C*) Flow cytometric analysis (FACS) of the sub-G (apoptotic) SCC2 cells. SCC2 cells were transfected with the indicated siRNA and exposed to 24–48 hours of hypoxia followed by staining with propidium iodide (PI). The amount of cells in sub-G1 was determined. There were no changes in the size of apoptotic sub-G1 phase population of siPHD3 samples relative to siScr control. (*D*) Quantification of the apoptotic sub G1 population in SCC2 cells. Three independent experiments are shown as folds vs. control (siScr).

As hypoxia is a known inducer of apoptosis we asked whether the reduction in cell amount was due to enhancement of hypoxia-induced apoptosis [39]. SCC2 cells were transfected with control and PHD3 siRNA followed by hypoxia for 6 to 30 hours. The cells were stained for cleaved caspase-3 which is activated during apoptosis and widely used as an apoptotic marker. Activation of caspase-3 was detected in a subpopulation of siPHD3 transfected cells as compared to control cells after 24-hour hypoxia (Fig. 4A) while most cells remained caspase-3 negative. Quantification demonstrated increased activation of caspase-3 by siPHD3 at early hypoxia (24-h) that was lost at later time points (Fig. 4B).

Caspase-3 activation in a subpopulation of siPHD3 cells could be suggestive for increased apoptosis. However, the increased caspase-3 cleavage does not necessarily lead to terminal apoptosis [40]. Therefore, we next used flow cytometry to further analyze the occurrence of apoptosis by studying the cell population in sub-G phase. The SCC2 cells were transfected with control or PHD3 siRNA and cultured in normoxia and hypoxia for 24 to 72 hours. As well established, the hypoxic exposure induced apoptosis in both control and siPHD3 cells. In control cells a 35% induction in apoptosis rate after 24-hour hypoxia was detected and after 72 hours the apoptosis rate had increased 2-fold (Fig. 4C, Fig. S3). However, when compared to the normoxic siScr control, the siPHD3 transfected cells demonstrated similar or even slightly reduced amount of cell in the sub-G phase (Fig. 4C, Fig. S3). When compared to siSCr controls at each time point, quantification of the apoptotic rate demonstrated small effect of siPHD3 on apoptosis that was insignificant (Fig. 4D). We concluded that although the exposure of SCC cells to PHD3 inhibition in hypoxia may activate caspase-3 in a subpopulation of cells this does not translate to major increase in apoptosis.

### PHD3 inhibition enhances hypoxia-induced block in G1 to S transition

**Figure 5:**
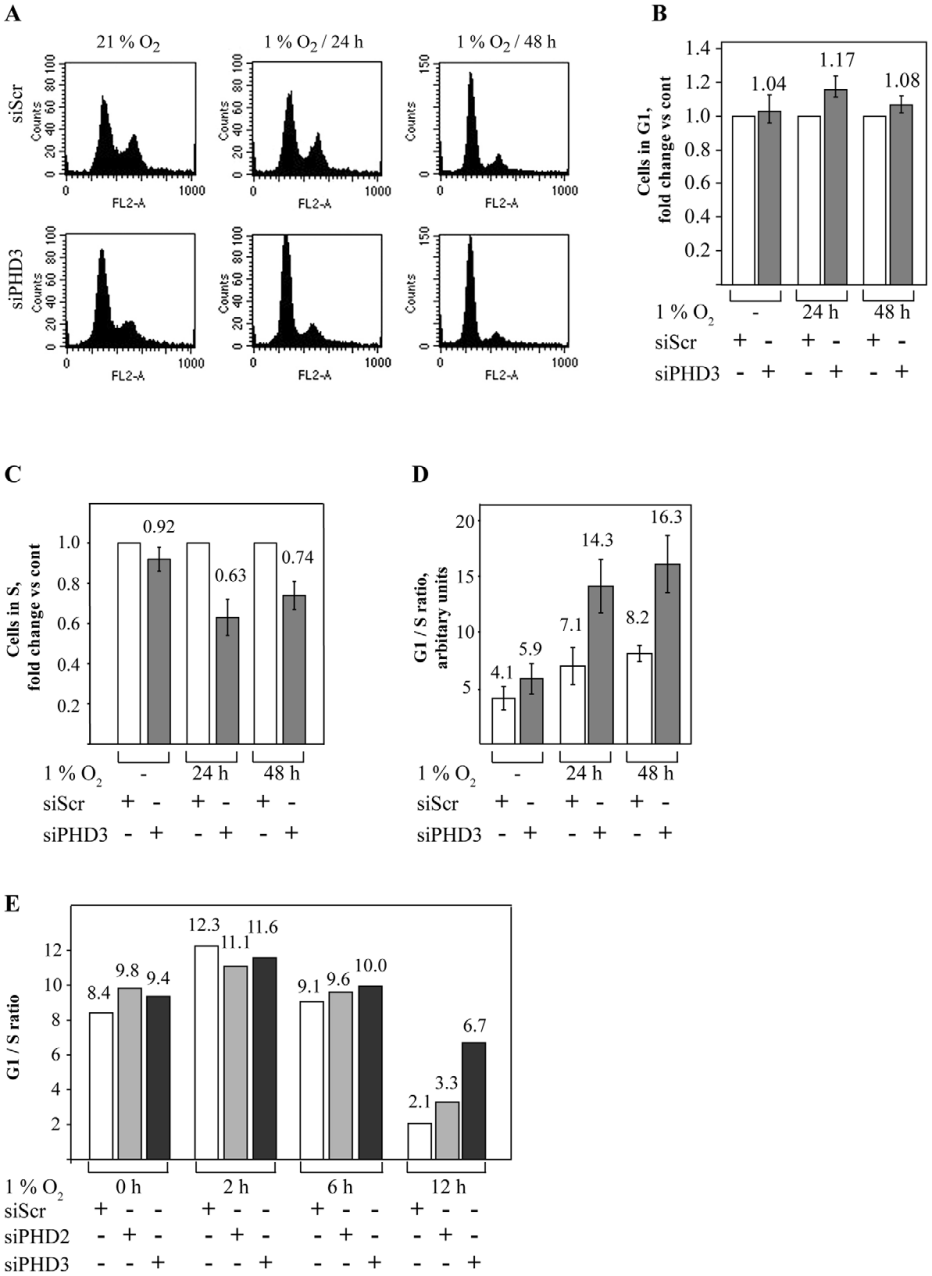
PHD3 inhibition causes a block in G1 to S-phase transition in hypoxia. (*A*) SCC2 cells were transfected with the indicated siRNAs and exposed to normoxia (21% O_2_) or hypoxia (1% O2) for 24 to 48 hours. Samples were and stained with PI and analyzed with FACS. The data was plotted as cell cycle histograms (FL2-A vs. counts). The cells exposed to PHD3 siRNA showed an increase in the size of G1 population and a decrease in the S and G2+M populations relative to control. The histograms show representative examples. (*B*) Quantification of cells in G1 phase after exposure to the indicated siRNAs and hypoxic treatments. Data from three independent experiments are shown as fold change vs. control (siScr). Means and SD are shown. (*C*) Quantification of cells in the S phase after exposure to the indicated siRNAs and hypoxic treatments. Data from three independent experiments are shown as fold change vs. control (siScr). Means and SD are shown. (*D*) Calculation of the relative sizes of the G1 to S phase from three independent experiments. (*E*) HeLa cells were treated with the indicated siRNAs followed by synchronization of the cells. After release the cells were exposed to hypoxia for the indicated time. Population sizes of the G1 and S phase cells were determined by FACS and the G1 to S relation was calculated (arbitrary units).

As the reduction in cell number could not be explained with increased apoptosis, we looked for possible defects in the cell cycle progression. SCC2 cells were transfected with either control or PHD3-targeted siRNA and incubated in normoxia and in hypoxia for 24 to 48 hours. Cell cycle was determined using flow cytometer with propidium iodide staining. As expected, hypoxic exposure led to an increase in the number of cells in G1 phase and a decrease in the cell population in S and M phases that peaked after 48-hour hypoxia indicating a block in G1 to S transition under hypoxia (Fig. 5A and Fig. S4). Noticeably, siPHD3 transfection led to further increase in the accumulation of cells in G1 (Fig. 5A and B). Accordingly, this was accompanied by a decrease in the amount of cells in the synthesis (S) phase. Quantification of the data from at least three independent experiments demonstrated constant increase in the G1 population under hypoxic PHD3 inhibition (Fig. 5B). Similarly, when compared to normoxic control, an approximately 10% increase in G1 was seen with PHD3 inhibition under hypoxia (Fig. S4A). In line with an increase in the G1, a marked decrease in the S phase with siPHD3 as compared to the siScr control at each time point was demonstrated under hypoxia (Fig. 5C, Fig. S4B). The percentual decrease in the S phase by siPHD3 transfection was approximately 35% and 25% at 24 and 48-hour hypoxia, respectively. Supporting reduced S-phase entry, a clear reduction of hypoxia-exposed cells in G2/M phase was also detected (Fig. 5A). The smaller increase in G1 population as compared to the reduction of S-phase reflects the fact that most cells at any given time reside in G1 phase. Noticeably, when the ratio between G1 and S phases was calculated, the difference between control and PHD3 inhibition was most evident illustrating a 2-fold increase in hypoxic siPHD3 cells as compared to control at 24- and 48-hour hypoxia (Fig. 5D). In line with previous reports on the effect of hypoxia on G1 to S transition, the difference in G1/S ratio showed 30% and 100% increase by hypoxia alone at 12- and 24-hours, respectively. This was further increased to 70% and 170% by siPHD3 exposure (Fig. S4C).

To study whether the siPHD3-induced block in G1 to S transition was specific for SCC cells we used HeLa cells that were synchronized and subsequently analyzed by flow cytometry at shorter timepoints. Hela cells were transfected with PHD3 and control siRNAs, synchronized with aphidicolin followed by exposure to hypoxia for 2 to 12 hours (Fig. S5) and calculation of G1 to S phase ratio. A marked increase in the G1 to S ratio in siPHD3 cells as compared to either siScr control or siPHD2-transfected cells was seen (Fig. 5E). The most notable difference was seen after 12-hour hypoxia when the G1 to S ratio in siPHD3 cells showed a 3-fold increase compared to controls (Fig. 5E). Together the data demonstrated that in hypoxic carcinoma cells PHD3 inhibition causes cell cycle arrest at the G1-S border.

### PHD3 depletion reduces the amount of hyperphosphorylated retinoblastoma protein in hypoxia

**Figure 6:**
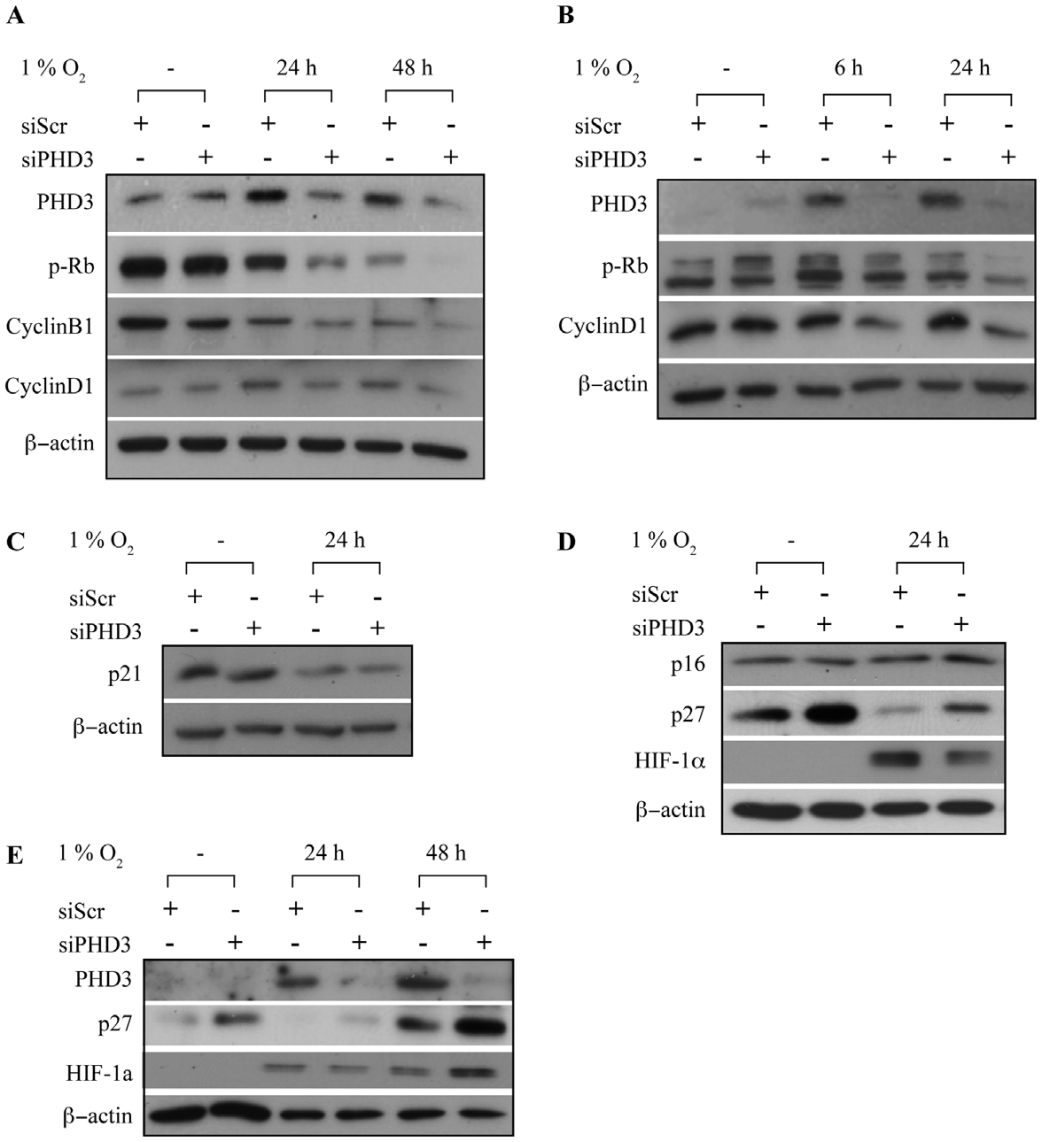
PHD3 inhibition reduces the amount of hyperphosphorylated Rb and increases p27 in hypoxia. (*A*) SCC2 cells were transfected with the indicated siRNAs and exposed to normoxia or hypoxia for 24 to 48 hours followed by western blot analysis of PHD3, phosphorylated Rb (p-Rb) and cyclin B1. (*B*) HeLa cells were transfected with the indicated siRNAs, synchronized and exposed to normoxia or hypoxia for 6 to 24 hours after release. PHD3, phosphorylated Rb (p-Rb) and cyclin D1 were analyzed from samples by western blotting. (*C*) SCC2 cells were transfected with the indicated siRNAs and exposed to normoxia or hypoxia for 24 hours followed by western blot analysis of p21(Cip1). (*D*) Cells transfected with the indicated siRNAs and exposed to normoxia or hypoxia for 24 hours followed by western blot analysis of p16 and p27. Hypoxia was monitored by HIF-1a expression. (*E*) Cells transfected with the indicated siRNAs and exposed to normoxia or hypoxia for 24 and 48 hours followed by western blot analysis of p27.

Hyperphosphorylation of retinoblastoma protein Rb (pRb) is required for the cell cycle to proceed from G1 to S phase [7], [8]. As we detected a block in the S-phase progression, we next asked whether the PHD3 inhibition would have an effect on the phosphorylation status of Rb. SCC2 cells transfected with control or PHD3 siRNAs followed by hypoxic exposure were analyzed for phosphorylated Rb at 6 and 48-hour timepoints (Fig. 6A). As expected, a marked decrease in the amount of pRb in SCC2 cells was detected under hypoxia as compared to normoxia. Importantly, PHD3 depletion strongly enhanced the hypoxic decrease already at 6 hours of hypoxia. At 48 hours the pRb level was further suppressed in siPHD3 transfected cells (Fig. 6A).

We next analyzed the expression of cyclins B and D. Cyclin D1 forms a complex with cyclin dependent kinases 4 and 6 (CDK4\6) which phosphorylate Rb and drive the cell cycle. Cyclin D1 expression is elevated during G1 to S transition [41]. Cyclin B1 is a regulatory subunit of cyclin-dependent kinase 1 (Cdk1) and is needed for G2 to M phase transition of cell cycle. Cyclin B1 expression serves as a switch for cells to proceed to mitosis. In accordance with the reduced G1 to S transition, we detected a decrease in cyclin D1 levels in hypoxic siPHD3-treated cells (Fig. 6A). Also a clear reduction in cyclin B1 expression was detected in cells exposed to hypoxia. The reduction was futher enhanced by PHD3 depletion (Fig. 6A). PHD3 depletion did not affect the expression of other cyclins such as cyclin A or cyclin E (not shown).

The expression analysis of cell cycle regulators was further performed in synchronized HeLa cells (Fig. 6B). In line with the data from SCC cells, a marked decrease in the amount of pRb in synchronized siPHD3-treated Hela cells was detected (Fig. 6B). In HeLa cells a decrease in cyclin D1 levels in siPHD3-treated cells was evident as well, while hypoxia per se showed little effect on cyclin D1 level. The suppression of Rb hyperphosphorylation with reduced expression of cyclins D1 and B1 in cells exposed to siPHD3 validates the effect of siPHD3 on cell cycle arrest seen in flow cytometric analyses.

The hypoxic cell cycle arrest has been reported to be p53 and p21(Cip1/CDKN1A) independent. In line with this, the knock-down of PHD3 did not lead to upregulation of p21 under hypoxia (Fig. 6C). We further studied the expression of two other cyclin-dependent kinase inhibitors, p16(INK4a) and p27(Kip1/CDKN1B) under hypoxic PHD3 inhibition. Similarly to p21, p16 levels remained unchanged under siPHD3 exposure. In striking contrast, the inhibition of PHD3 led to clear upregulation of p27, which is known to arrest cell cycle at G1-phase. This was evident both in normoxia and hypoxia (Fig. 6D). Interestingly, the p27 level was reduced by hypoxia at 24 hour time point but increased at 48 hours as compared to normoxia (Fig. 6E). Taken together the data indicate that PHD3 is required for cell cycle to progress from G1 to S-phase under hypoxia and that p27 may mediate the effect.

## Discussion

PHD3 is upregulated by hypoxia and the elevated levels remain in prolonged hypoxia enabling long-term regulation of hypoxic cell fate [28], [29], [30]. Some PHD3 hydroxylation activity is preserved at least under moderate hypoxia (1% O_2_) where it has been suggested to desensitize and prevent excessive HIF-alpha activity [32], [34]. Here we have studied the hypoxic function of PHD3 in carcinoma cells. First we demonstrated that PHD3 is elevated in human cancers of the head and neck region. The increase of PHD3 during carcinogenesis suggested that it may facilitate tumor progression. Supporting this, the inhibition of PHD3 expression in cells derived from SCC led to reduced cell survival under hypoxia. Importantly, we demonstrated that the inhibition of PHD3 causes cell cycle arrest of carcinoma cells in hypoxia. The arrest was pinpointed to the G1/S boundary and was accompanied by a block in Rb hyperphosphorylation as well as reduced expression of the cyclins D1 and B1. Together the data suggests that hypoxic upregulation of PHD3 in HNSCC is necessary for cell survival in hypoxia by allowing cell cycle to proceed from G1 to S-phase.

Hypoxia is a well-characterized environmental factor that halts cell cycle and cell proliferation [5], [6]. Since energy availability from oxidative phosphorylation is reduced in hypoxia, it is likely that cell cycle arrest is to prevent excessive cell death under reduced oxygen [42]. The hypoxic cell cycle arrest occurs at G1 preventing cells from proceeding to S-phase where they would be most susceptible to cell death. In keeping with this, hypoxia is known to result in Rb hypophosphorylation and reduced cyclin D1 expression [43], [44]. However, during the growth of carcinomas a subpopulation of cells need to escape the cell cycle regulation in order to maintain cell growth also under low oxygen tension. The molecular mechanisms that underlie the hypoxia-induced cell cycle arrest or the mechanisms that can release the arrest in carcinoma cells are not fully understood. Our work directly links the primary oxygen sensors to the hypoxic cell cycle arrest as well as Rb hypophosphorylation and reduced cyclin D1 expression. Out of the three cyclin-dependent kinase inhibitors studied, p27 was the only one the expression of which displayed changes upon PHD3 inhibition. p27 has several functions in regulating cell cycle [45]. Expression of p27 at G1 phase inhibits cyclin E/A – CDK2 blocking the transcription of genes that are required for the G1 to S transition. In particular under adverse conditions p27 is also known to inhibit the activation of cyclin D – CDK4 complex and cause a block in S-phase transition. This is well in line with reduced cyclin D1 expression and G1 arrest seen by PHD3 inhibition. Therefore, it is feasible that p27 mediates the hypoxic effects of PHD3 on cell cycle. Whether PHD3 affects the transcription or protein degradation of p27 remains to be investigated. Moreover, it is not clear whether the hypoxic block requires p27 cell cycle inhibitor activity [10], [11]. This may be context-dependent and also vary depending on the length of hypoxia. Our data indicate that p27 is first suppressed under hypoxia but reactivated upon longer exposure.

Under hypoxia PHD3 expression is activated by HIF [28], [30], [31]. In turn, HIF-α is the main known enzymatic target of PHD3. Whether the downstream effects of PHD3 on cell survival are mediated by HIF remain elusive. Given the large body of evidence that HIF functions as a protective factor for hypoxic cells it is difficult to see how the knockdown of an HIF inhibitor PHD3 could cause increased cell death. However, one needs to keep in mind that PHD3 is likely to fine tune the HIF-α level in hypoxia where excessive HIF accumulation might shift the cell fate decision towards cell death. Experimentally it will be challenging to study the possible HIF-mediated effects on cell cycle under PHD3 inhibition, as the effect of PHD3 in hypoxia may be to retain HIF level within a narrow range that can support cell cycle progression. Reaching such level in a large cell population using simultaneous HIF and PHD3 inhibition will be challenging. Supporting the idea that the functions are not mediated by HIF, the rescue experiments suggested that PHD3 hydroxylase activity is not required for cell survival supporting function. This is not completely surprising since a number of hydroxylase activity independent functions have been ascribed for the PHD family members [23], [46]. Interestingly also, the experiments suggested that even when only a fraction of the cells express PHD3, this is reflected in a survival benefit for a larger cell population, suggesting a bystander effect.

Several studies have indicated that when overexpressed in normoxia, PHD3 can activate apoptotic cell death in neuronal cells [24], [25], [27], [47]. Such cell death induced by PHD3 has also been reported in some carcinoma cells [26], [48]. However, similarly to our work, more recent reports have also indicated that under hypoxia PHD3 may in fact protect cancer cells from cell death induced either by hypoxia or other apoptotic factors [49]. This implies that PHD3 has a dualistic function in regulating cell fate decisions that depend at least on cell-type and oxygen availability but probably also on other interacting factors. For example, a recent study suggests that inhibition of PHD3 in hypoxic carcinoma cells lacking PRP19 may reduce apoptosis [50]. In the present study we detected activation of caspase-3 in a subpopulation of siPHD3 transfected cells under hypoxia. Initially this could have been interpreted as an increase in the apoptosis rate. However, it has been well documented that caspase-3 activation does not necessarily lead to terminal apoptosis [40]. In keeping with this, we were unable to detect any significant increase of terminal apoptosis in siPHD3-exposed cells by flow cytometry. It needs to be underlined that these analyses are internally controlled by reduced S-phase entry. The data strongly argues against apoptosis as the main cause of reduced cell survival by PHD3 inhibition in hypoxic squamous carcinoma cells.

PHD1 has recently also been shown to regulate cell cycle progression and cyclin D1 expression [51]. This was shown in estrogen-dependent breast carcinomas where loss of PHD1 led to decrease in cyclin D level and subsequently impaired cell proliferation. In the current work we focused on the hypoxia-induced PHD isoforms PHD2 and -3. In glioblastoma cells the inhibition of either PHD2 or PHD3 was protective against hypoxia and staurosporine-induced cell death [49]. In SCC cells however, PHD2 inhibition demonstrated far less cell death or effects on the cell cycle as compared to PHD3. Together the data suggests cell-type specific effects of the PHD isoforms on cell viability and cell cycle regulation. PHD3-specific inhibition could be a feasible mean to block the hypoxia-induced cell survival at least in squamous cell carcinomas.

## Materials and Methods

### Patient samples

Tumor samples were collected, after informed written consent, from surgically removed oral and oropharyngeal HNSCC in Turku University Hospital, Finland. Normal tissue samples used as a control were collected from patients undergoing uvulopalato-pharyngoplasty. Samples were from both genders and the age of the patients ranged from 29 to 87 years. All procedures in this study were approved by the National Authority for Medicolegal Affairs and by the ethics committee of Turku University Hospital. All data were analyzed anonymously. The patient material has been described in detail elsewhere [52], [53]. For database studies Oncomine™ was used [36].

### Cell culture and transfections

HeLa cells were obtained from the American Type Culture Collection, Rockville, MD, USA. The cancer patient-derived HNSCC cell lines UT-SCC2, UT-SCC7 and UT-SCC9 used in this study have been at the University of Turku and described previously [35]. Cells were cultured in DMEM, supplemented with 10% FCS, penicillin-streptomycin and L-glutamine. The SCC cells were also supplemented with nonessential amino acids (Sigma-Aldrich). Cells were cultured in humidified air conditioning 5% CO_2_ at 37°C. For hypoxia treatments air was replaced by nitrogen to reach 1% oxygen in a hypoxia workstation (Invivo_2_).

For siRNA experiments, double stranded siRNA oligonucleotides were used at 200 nM final concentration. Transfections were performed with Oligofectamine (Invitrogen) following the manufacturer's protocol. The siRNAs (MWG Biotechnology) used were: siPHD2 5′-GACGAAAGCCAUGGUUGCUUG (dTdT)-3′; siPHD3 5′-GUCUAAGGCAAUGGUGGCUUG(dTdT)-3′; siPHD3 off-target 5′-GUC UAAGGCAUAGGUGGCUUG(dTdT)-3′ and non-target (scr) siRNA 5′-CCUACAUCCCGAUCG AUGAUG(dTdT)-3′.

For some of the cell cycle experiments the cells were synchronized using aphidicolin at 1 µg/ml. 18 to 22 hours after incubation the cell cycle was released by washing the cells with PBS three times and adding fresh media to the cells.

### mRNA, western blot analysis and antibodies

RNA was extracted using RNeasy mini kit (Qiagen). Q-RT-PCR (Taqman) cDNA was prepared with M-MMLV RT (H^−^) (Promega) and NTP-mix (Fermentas) at 0,5 mM and analyzed according to manufacturer's instructions (Applied Biosystems). Taqman primers (Eurogentec) used were: PHD3 fwd 5′ – CGAAGTGCAGCCCTCTTACG – 3′; PHD3 rev 5′ – TTTTGGCTTCTGCCCTTTCTT – 3′ and PHD3 probe (Eurogentec) 5′ – AACCAGATATGCTATGACTGTCTGGTACTTTGATGCT – 3′. The expressions were normalized against EF-1α.

For protein analysis cells were harvested in SDS-Triton lysis buffer (50 mM Tris-HCl pH 7.5, 150 mM NaCl, 0.5% Triton X-100, 5% glycerol, 1% SDS, 1 mM Na_3_VO_4_, 1 mM phenylmethylsulphonyl fluoride and 10 mM NaF,). Protein concentrations were measured with Bio-Rad DC protein assay before addition of 6×SDS buffer. Proteins were detected by Western blotting and enhanced chemiluminescence with specific antibodies: active caspase 3 (G748A, Promega, 1∶500), cyclin B1 (sc-752, Santa Cruz Biotechnology, 1∶2000), cyclin D1 (sc-246, Santa Cruz Biotechnology, 1∶2000), HIF-1alpha (610959, BD Transduction Laboratories, 1∶3000), PHD2 (NB 100–137, Novus Biologicals, 1∶3000), PHD3 (NB 100–139, Novus Biologicals, 1∶2000) and Ac-74 β-actin antibody (Sigma-Aldrich, 1∶4000). Secondary anti-mouse-HRP and anti-rabbit-HRP (DAKO) antibodies were used at 1∶10 000.

### Cell proliferation, imaging and immunocytochemistry

For BrdU proliferation assay the cells were transfected with corresponding siRNA and plated on 96-well plates (8 wells per siRNA). Plates were incubated in normoxia or in hypoxia for 48 hours. BrdU was added and incubated for 4 hours. The cell proliferation was measured with colorimetric ELISA, BrdU assay (Roche Diagnostics). BrdU assay was performed according to manufacturer's instructions.

For cell counting the cell nuclei cells were fixed with PTEMF fixing solution (100 mM PIPES pH 6.8, 10 mM EGTA, 1 mM MgCl_2_, 0,2% Triton X-100 and 4% formaldehyde) and stained with the nuclear stain Hoechst 33342 (Invitrogen). Optical fields of cells were imaged with Zeiss Lumar V12 fluorescence stereo microscope (Carl Zeiss) and the number of nuclei per optical field was calculated using ImageJ software (NIH, USA) nucleus calculator option. Experiments were done as parallel treatments and each experiment was repeated at least three times. For detection of active caspase 3 the cells were stained with caspase 3 antibody described earlier.

### Flow cytometry

Cell cycle and apoptosis were detected with flow cytometer (BD FACSCalibur, BD Biosciences). The samples were collected by trypzination, washed and fixed with 70% ethanol in −20°C for 24 h. The fixing solution was removed by centrifugation and nuclei were washed with PBS. Staining was done in RT with propidium iodide (PI) (10 µg/ml) in PBS with 0,1% Tween. The cell cycle histograms were analyzed using BD CellQuest Pro software (BD Biosciences).

### Statistics

For statistical analysis Spearman correlation and two-tailed Student's t-test were used.

## Supporting Information

Figure S1 **Hypoxia induces the expression of PHD2 but not PHD1 in UT-SCC.** Five different primary human head and neck squamous cell carcinoma-derived cell lines (SCC) were cultured in normoxia or in hypoxia for 6 and 48 hours. The cells were exposed to normoxia or hypoxia for 6 and 48 hours. The PHD mRNA expression was detected and quantified by Q-RT-PCR. (*A*) PHD1 mRNA levels reduced during prolonged hypoxic exposure whereas (*B*) the PHD2 levels were induced in short hypoxia. (*C*) PHD3 mRNA levels under siPHD3 exposure in normoxia (left-hand panel) and hypoxia (right-hand panel). Please note the different scale for normoxia and hypoxia.
(TIF)

Figure S2 **Expression of transfected EGFP, PHD3-EGFP and PHD3R206K-EGFP (** **Fig. 3** **) studied by confocal microscopy.**
(TIF)

Figure S3 **Hypoxia-activated apoptosis is not enhanced by PHD3 inhibition.** The apoptosis rate was determined by flow cytometer at the indicated time points. The hypoxic exposure increased the size of apoptotic sub G1 population in both control (siScr) transfected and siPHD3 cells.
(TIF)

Figure S4 **PHD3 inhibition causes a block in G1 to S transition under hypoxia.** (*A*) Quantification of cells in G1 phase after exposure to the indicated siRNAs and hypoxia. The data from figure 4 is calculated as fold change vs. normoxic control. Data from three independent experiments with means and SD are shown. (*B*) Quantification of cells in S phase after exposure to the indicated siRNAs and hypoxia. The data from figure 4 is calculated as fold change vs. normoxic control. Data from three independent experiments with means and SD are shown. (*C*) Hypoxic G1 to S phase block is enhanced by PHD3 inhibition. SCC2 cells were transfected with the indicated siRNAs and exposed to normoxia (21% O_2_) or hypoxia (1% O_2_) for 24 to 48 hours. Samples were and stained with PI and analyzed with FACS. Calculation of the relative sizes of the G1 to S phase from three independent experiments. The data are shown as increase normoxic controls.
(TIF)

Figure S5 **The effect of aphidicolin on cell cycle progression under normoxia and hypoxia.** (*A*) Aphidicolin causes a cell cycle block which is released immediately after medium change. The cell cycle was blocked by aphidicolin treatment for 20 to 22 hours. The cell cycle was released by washing with PBS and by adding fresh media on the cells. The progression of the cell cycle was followed by flow cytometry in normoxia (21% O_2_) and hypoxia (1% O_2_). The curves illustrate the release of cell cycle inhibition (0 h) at S phase over 24 hours. (*B*) The effect of aphidicolin and release for G1 phase for normoxia (21% O_2_) and hypoxia (1% O_2_).
(TIF)

Table S1 **Selected studies extracted from Oncomine database investigating PHD3 mRNA expression in diverse cancers vs. matching normal tissue.**
(DOC)
