# Supplementary figures and images for: Prolyl Hydroxylase PHD3 Enhances the Hypoxic Survival and G1 to S Transition of Carcinoma Cells
Source: PLoS One. 2011 Nov 8;6(11):e27112. doi: 10.1371/journal.pone.0027112 (PMC3210766; doi:10.1371/journal.pone.0027112)

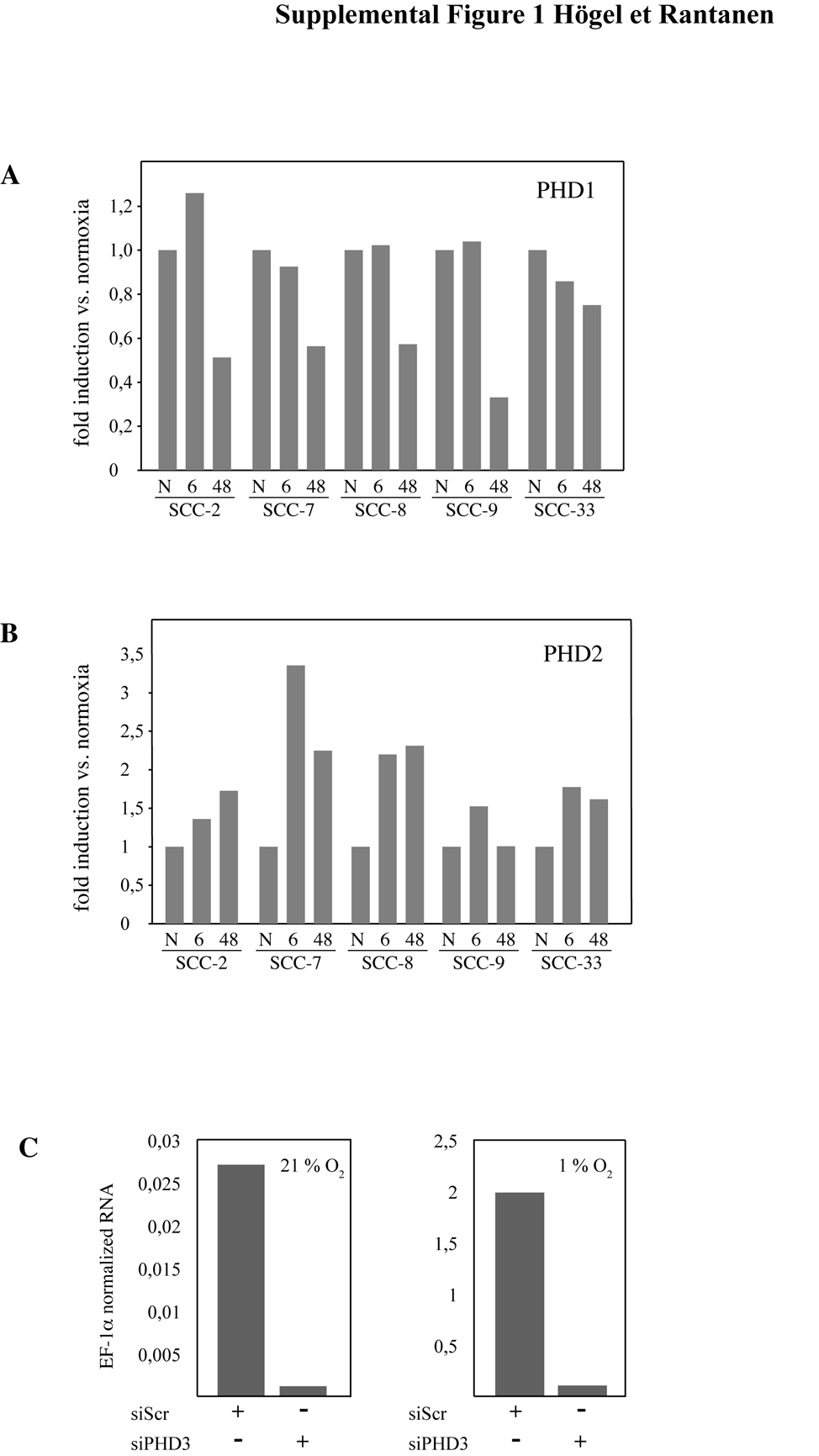

Supplement: Figure S1 — Hypoxia induces the expression of PHD2 but not PHD1 in UT-SCC. Five different primary human head and neck squamous cell carcinoma-derived cell lines (SCC) were cultured in normoxia or in hypoxia for 6 and 48 hours. The cells were exposed to normoxia or hypoxia for 6 and 48 hours. The PHD mRNA expression was detected and quantified by Q-RT-PCR. (A) PHD1 mRNA levels reduced during prolonged hypoxic exposure whereas (B) the PHD2 levels were induced in short hypoxia. (C) PHD3 mRNA levels under siPHD3 exposure in normoxia (left-hand panel) and hypoxia (right-hand panel). Please note the different scale for normoxia and hypoxia. (TIF) [file pone.0027112.s001.tif]

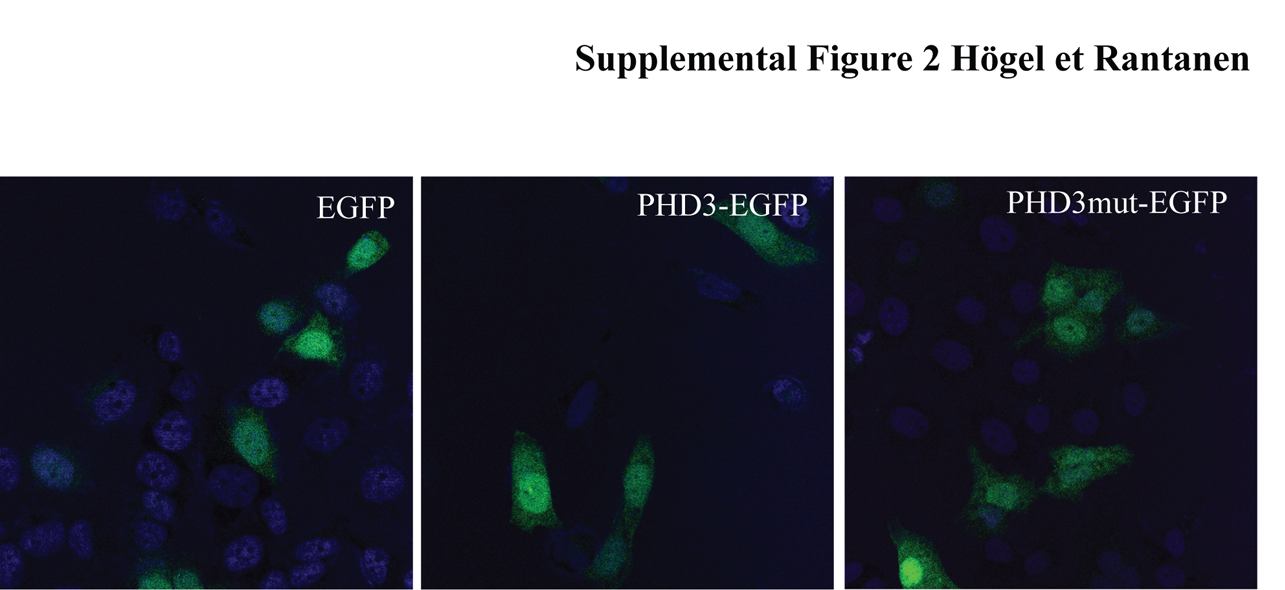

Supplement: Figure S2 — Expression of transfected EGFP, PHD3-EGFP and PHD3R206K-EGFP ( Fig. 3 ) studied by confocal microscopy. (TIF) [file pone.0027112.s002.tif]

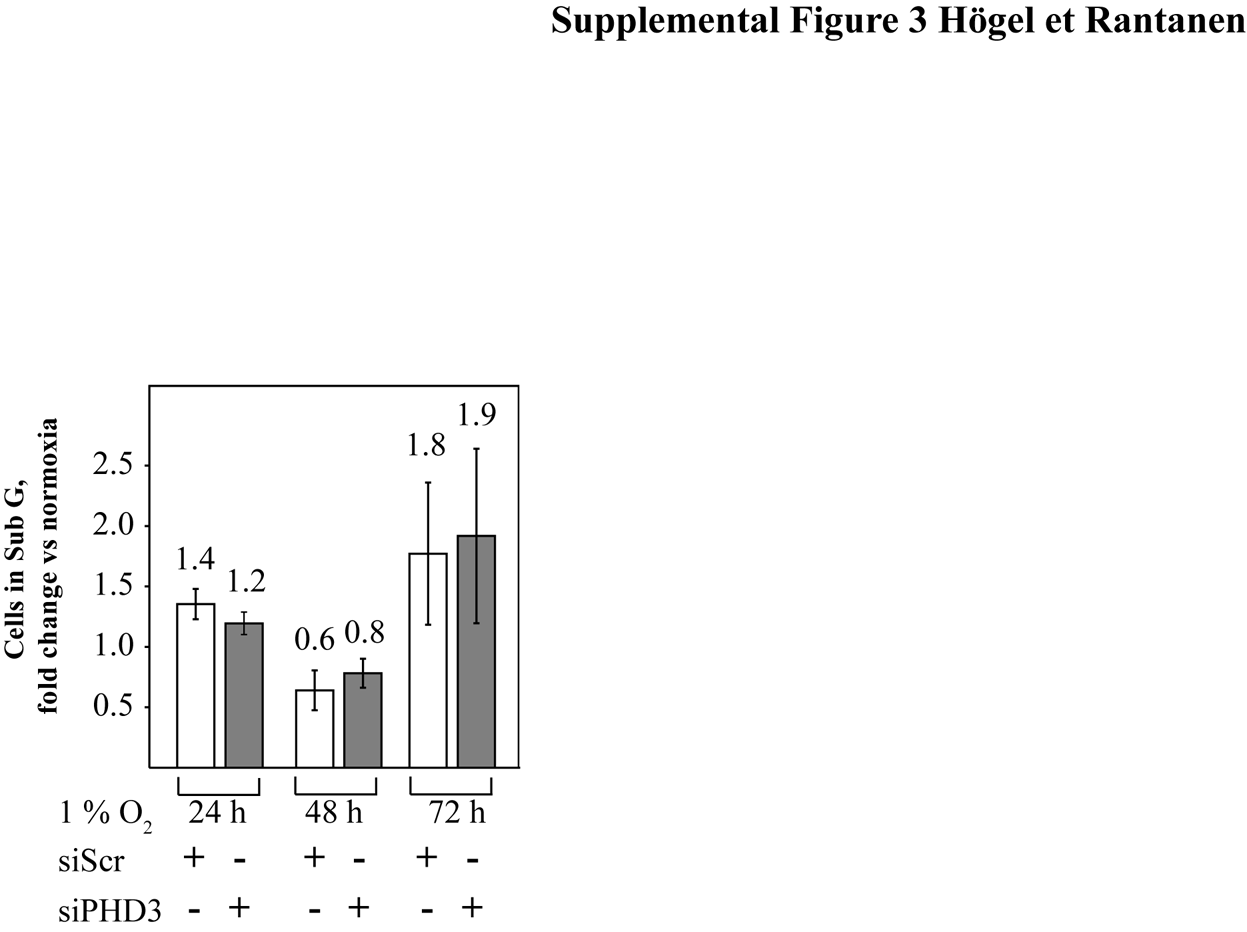

Supplement: Figure S3 — Hypoxia-activated apoptosis is not enhanced by PHD3 inhibition. The apoptosis rate was determined by flow cytometer at the indicated time points. The hypoxic exposure increased the size of apoptotic sub G1 population in both control (siScr) transfected and siPHD3 cells. (TIF) [file pone.0027112.s003.tif]

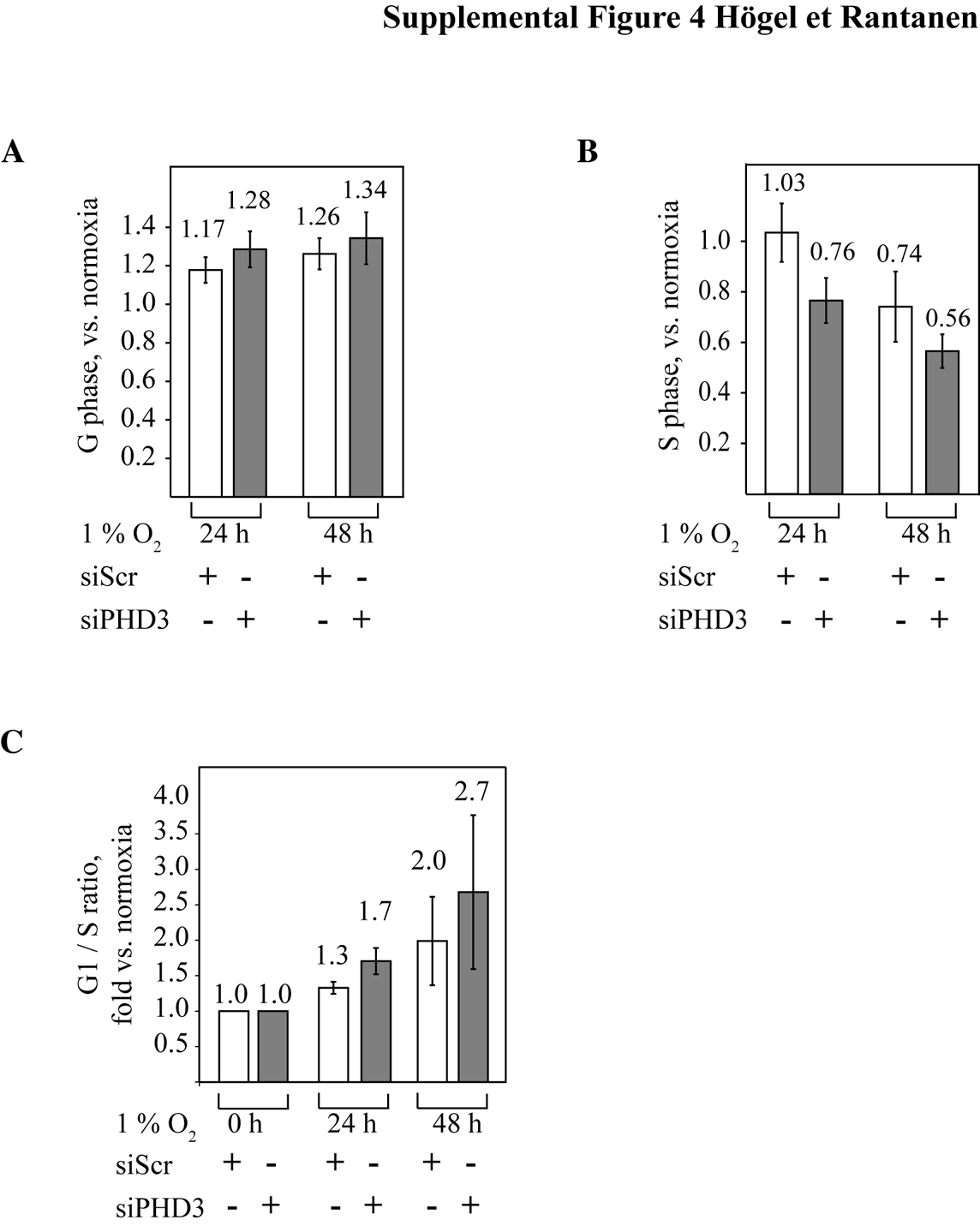

Supplement: Figure S4 — PHD3 inhibition causes a block in G1 to S transition under hypoxia. (A) Quantification of cells in G1 phase after exposure to the indicated siRNAs and hypoxia. The data from figure 4 is calculated as fold change vs. normoxic control. Data from three independent experiments with means and SD are shown. (B) Quantification of cells in S phase after exposure to the indicated siRNAs and hypoxia. The data from figure 4 is calculated as fold change vs. normoxic control. Data from three independent experiments with means and SD are shown. (C) Hypoxic G1 to S phase block is enhanced by PHD3 inhibition. SCC2 cells were transfected with the indicated siRNAs and exposed to normoxia (21% O2) or hypoxia (1% O2) for 24 to 48 hours. Samples were and stained with PI and analyzed with FACS. Calculation of the relative sizes of the G1 to S phase from three independent experiments. The data are shown as increase normoxic controls. (TIF) [file pone.0027112.s004.tif]

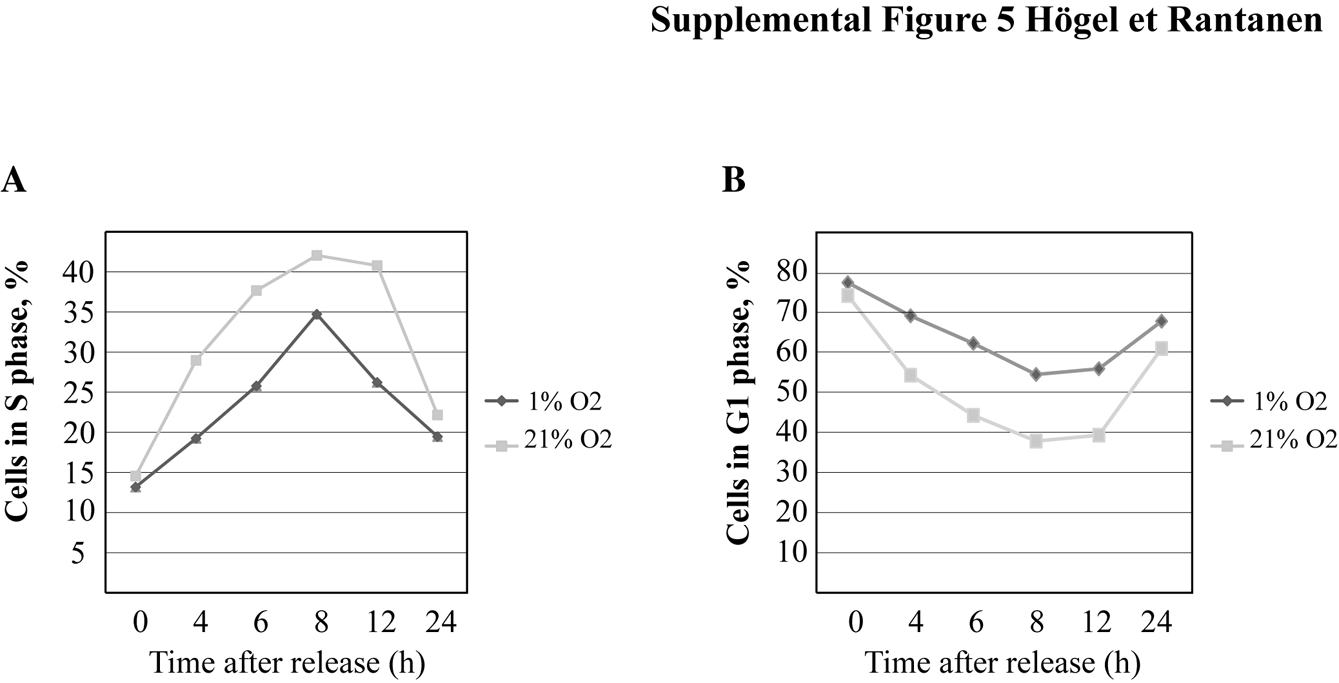

Supplement: Figure S5 — The effect of aphidicolin on cell cycle progression under normoxia and hypoxia. (A) Aphidicolin causes a cell cycle block which is released immediately after medium change. The cell cycle was blocked by aphidicolin treatment for 20 to 22 hours. The cell cycle was released by washing with PBS and by adding fresh media on the cells. The progression of the cell cycle was followed by flow cytometry in normoxia (21% O2) and hypoxia (1% O2). The curves illustrate the release of cell cycle inhibition (0 h) at S phase over 24 hours. (B) The effect of aphidicolin and release for G1 phase for normoxia (21% O2) and hypoxia (1% O2). (TIF) [file pone.0027112.s005.tif]
