# Supplementary material for: Prolyl Hydroxylase PHD3 Enhances the Hypoxic Survival and G1 to S Transition of Carcinoma Cells
Source: PLoS One. 2011 Nov 8;6(11):e27112. doi: 10.1371/journal.pone.0027112 (PMC3210766; doi:10.1371/journal.pone.0027112)
Supplement: Table S1 — Selected studies extracted from Oncomine database investigating PHD3 mRNA expression in diverse cancers vs. matching normal tissue. (DOC) [file pone.0027112.s006.doc]

| **Dataset normal vs cancer** | **location** | **type** | **n, normal** | **n, cancer** | **oe, folds** | **oe, avg** | **oe, median** | **p value** |
| --- | --- | --- | --- | --- | --- | --- | --- | --- |
|  |  |  |  |  |  |  |  |  |
| **RENAL clear cell carcinoma** |  |  | **35** | **104** | **5,0 - 36,2** | **17,48** | **12,162** |  |
| Yusenko Renal | renal | Clear cell RCC | 5 | 26 | 12,162 |  |  | 1,40E-18 |
| Beroukhim Renal | renal | Non-her. Clear celRCC | 11 | 32 | 21,965 |  |  | 3,78E-15 |
|  | renal | Hered. Clear cel RCC |  | 27 | 36,176 |  |  | 9,50E-19 |
| Gumz Renal | renal | Clear cell RCC | 10 | 10 | 12,101 |  |  | 1,75E-10 |
| Lenburg Renal | renal | Clear cell RCC | 9 | 9 | 5,007 |  |  | 6,71E-06 |
|  |  |  |  |  |  |  |  |  |
| **LUNG adenocarcinoma** |  |  | **88** | **136** | **1,7 - 2,0** | **1,91** | **1,969** |  |
| Su Lung | lung | lung adenocarcinoma | 30 | 27 | 2,043 |  |  | 1,49E-05 |
| Landi Lung | lung | lung adenocarcinoma | 49 | 58 | 1,654 |  |  | 6,38E-13 |
| Yamagata Lung | lung | lung adenocarcinoma | 3 | 9 | 2,036 |  |  | 0,056 |
| Garber Lung | lung | lung adenocarcinoma | 6 | 42 | 1,902 |  |  | 2,62E-04 |
|  |  |  |  |  |  |  |  |  |
| **LUNG other carcinomas** |  |  | **14** | **46** | **1,8 - 3,4** | **2,55** | **2,382** |  |
| Yamagata Lung | lung | large lung cell carc | 3 | 5 | 3,397 |  |  | 0,01500 |
| Yamagata Lung | lung | squamous cell lung canc |  | 11 | 2,403 |  |  | 0,03300 |
| Garber Lung |  | squamous cell lung canc | 6 | 16 | 2,1 |  |  | 0,00200 |
| Garber Lung |  | large lung cell carc |  | 4 | 3,173 |  |  | 0,01200 |
| Garber Lung |  | small cell lung carc |  | 5 | 2,361 |  |  | 0,04300 |
| Wachi Lung | lung | squamous cell lung canc | 5 | 5 | 1,863 |  |  | 0,00900 |
|  |  |  |  |  |  |  |  |  |
| **BREAST ductal carcinoma** |  |  | **17** | **215** | **1,6 - 3,2** | **2,49** | **2,6365** |  |
| Sorlie Breast |  | ductal breast carcinoma | 4 | 63 | 2,49 |  |  | 0,02200 |
| Sorlie Breast 2 | breast | ductal breast carcinoma | 3 | 76 | 3,153 |  |  | 0,02700 |
| Perou Breast | breast | ductal breast carcinoma | 3 | 36 | 2,783 |  |  | 0,06000 |
| Richardson Breast 2 | breast | ductal breast carcinoma | 7 | 40 | 1,553 |  |  | 0,02500 |
|  |  |  |  |  |  |  |  |  |
| **BREAST other carcinomas** |  |  | **10** | **11** | **1,5 - 2,4** | **2,22** | **2,421** |  |
| Perou Breast | breast | lobular breast carcinoma | 3 | 4 | 2,739 |  |  | 0,06900 |
| Sorlie Breast 2 | breast | lobular breast carcinoma | 3 | 5 | 2,421 |  |  | 0,05100 |
|  | breast | fibroadenoma | 4 | 2 | 1,506 |  |  | 0,16200 |
|  |  |  |  |  |  |  |  |  |
| **LIVER, hepatocellular carcinoma** |  |  | **77** | **144** | **2,0 - 3,6** | **2,35** | **1,955** |  |
| Chen Liver | liver | hepatocellular carcinoma | 67 | 92 | 1,955 |  |  | 5,460E-08 |
| Wurmbach Liver | liver | hepatocellular carcinoma | 10 | 35 | 1,55 |  |  | 0,01000 |
| Wurmbach Liver | liver | liver cell dysplasia |  | 17 | 3,55 |  |  | 2,930E-08 |
|  |  |  |  |  |  |  |  |  |
| **PANCREAS (adenocarcinoma)** |  |  | **90** | **141** | **1,8 - 5,4** | **2,99** | **2,164** |  |
| Segara Pancreas | pancreas | pancreatic carcinoma | 6 | 11 | 1,814 |  |  | 0,00008 |
| Lacobuzio_Donahue Pacreas 2 | pancreas | pancreatic adenocarc | 4 | 31 | 3,455 |  |  | 0,00022 |
| Pei pancreas | pancreas | pancreatic carcinoma | 16 | 36 | 5,395 |  |  | 1,680E-09 |
| Ishikawa Pancreas | pancreas | pancreatic ductal adenocarc | 25 | 24 | 2,117 |  |  | 0,01800 |
| Badea Pancreas | pancreas | pancreatic ductal adenocarc | 39 | 39 | 2,164 |  |  | 0,00002 |
|  |  |  |  |  |  |  |  |  |
| **BLADDER** |  |  | **17** | **71** | **1,2 - 2,1** | **0,92** | **1,196** |  |
| Blaveri Bladder 2 | bladder | superficial bladder cancer | 3 | 24 | 2,094 |  |  | 0,00056 |
| Blaveri Bladder 2 | bladder | infiltrating bladder urothelial carcinoma |  | 42 | 1,427 |  |  | 0,02200 |
| Dyrskjot Bladder 3 | bladder | stage 0's bladder urothelial carc. | 14 | 5 | 1,196 |  |  | 0,04500 |
|  |  | superficial bladder cancer |  | 28 | 1,028 |  |  | 0,24600 |
| Dyrskjot Bladder 3 | bladder | infiltrating bladder urothelial carcinoma |  | 13 | -1,156 |  |  | 0,87800 |
|  |  |  |  |  |  |  |  |  |
| **OVARIAN cacncer** |  |  | **4** | **99** | **1,3 - 1,6** | **1,38** | **1,347** |  |
| Hendrix Ovarian | ovarian | ovarian muc. adenoc. | 4 | 13 | 1,54 |  |  | 2,440E-06 |
|  | ovarian | ovarian serous adenoc. |  | 41 | 1,365 |  |  | 1,800E-06 |
|  | ovarian | ovarian endometrioid adenocarc. |  | 37 | 1,329 |  |  | 0,00001 |
|  | ovarian | ovarian clear cell adenocarc. |  | 8 | 1,294 |  |  | 0,00200 |
|  |  |  |  |  |  |  |  |  |
| **BRAIN cancer** |  |  |  |  |  |  |  |  |
| Lee Brain | brain | glioblastoma | 3 | 22 | 2,814 |  |  | 0,00057 |
|  |  |  |  |  |  |  |  |  |
| **SARCOMA** |  |  | **9** | **118** | **1,2 - 1,9** | **1,53** | **1,5305** |  |
| Barretina Sarcoma | smooth muscle | leiomyosarcoma | 9 | 26 | 1,884 |  |  | 6,200E-06 |
|  | fat cells | liposarcoma |  | 92 | 1,177 |  |  | 0,02400 |
|  |  |  |  |  |  |  |  |  |
| **COLON cancer** |  |  | **33** | **150** | **1,2** | **1,24** | **1,235** |  |
| Ki colon | colon | colon adenocarcinoma | 28 | 50 | 1,225 |  |  | 0,01900 |
| Kaiser Colon | colon | colon mucinous adenocarc | 5 | 100 | 1,245 |  |  | 0,00300 |
|  |  |  |  |  |  |  |  |  |
| **TESTIS cancer** |  |  | **6** | **82** | 1,8 - 2,1 | **1,83** | **1,769** |  |
| Korkola Seminoma |  | embryonal carcinoma | 6 | 15 | 1,905 |  |  | 2,36E-08 |
|  |  | teratoma |  |  | 2,088 |  |  | 5,16E-07 |
|  |  | mixed germ cell tumor |  | 45 | 1,637 |  |  | 1,73E-07 |
|  |  | seminoma |  | 12 | 1,769 |  |  | 1,27E-04 |
|  |  | yolk sac tumor |  | 10 | 1,769 |  |  | 9,00E-03 |
|  |  |  |  |  |  |  |  |  |
| **PROSTATE cancer** |  |  |  |  |  |  |  |  |
| Liu prostate | prostata | prostate carcinoma | 13 | 44 | 1,033 |  |  | 0,03 |
